# Impact of CancelRx on discontinuation of controlled substance prescriptions: an interrupted time series analysis

**DOI:** 10.1186/s12911-022-01779-9

**Published:** 2022-02-25

**Authors:** Taylor L. Watterson, Jamie A. Stone, Aaron Gilson, Roger Brown, Ka Z. Xiong, Anthony Schiefelbein, Edmond Ramly, Peter Kleinschmidt, Michael Semanik, Lauren Craddock, Samantha I. Pitts, Taylor Woodroof, Michelle A. Chui

**Affiliations:** 1 University of Wisconsin-Madison School of Pharmacy, 777 Highland Avenue, 2511 Rennebohm Hall, Madison, WI 53704 USA; 2 University of Wisconsin-Madison School of Nursing, Madison, WI USA; 3 Wisconsin Department of Health Services, Madison, WI USA; 4 University of Wisconsin School of Medicine and Public Health, Madison, WI USA; 5 University of Wisconsin-Madison College of Engineering, Madison, WI USA; 6 SSM Health Dean Medical Group, Madison, WI USA; 7 Johns Hopkins Medicine, Baltimore, MD USA

**Keywords:** Health information technology, Interrupted time series, Controlled substances, Opioid epidemic, Prescriptions

## Abstract

**Background:**

Prescription opioid misuse is a serious national crisis; in 2018 the top drugs involved in prescription overdose deaths included pain medications (opioids), benzodiazepines, and stimulants. Health information technology (health IT) provides a means to address this crisis through technologies that streamline the prescribing and discontinuation process. CancelRx is a health IT function that communicates when medications, such as controlled substances, are discontinued at the clinic and therefore should not be filled at the pharmacy. Prior to CancelRx, the communication of discontinued medications was a manual process, requiring the patient or a clinic staff member to personally contact the pharmacy to inform them of the change. The objective of this study was to assess how controlled substance medication discontinuations were communicated over time, before and after the implementation of CancelRx.

**Methods:**

Secondary data from a midwestern academic health system electronic health record and pharmacy platform were collected 12-months prior to CancelRx implementation and for 12-months post implementation. The study utilized an interrupted time series analysis (ITSA) to capture the percentage of controlled substance medications that were discontinued in the clinic’s electronic health record and discontinued in the pharmacy’s dispensing software. The ITSA plotted the percentage of successful discontinuation messages over time, particularly after the health system’s implementation of CancelRx, a novel technology.

**Results:**

After CancelRx implementation there was an immediate (change = 77.7 percentage point) and significant (p < 0.001) increase in the number of controlled substance medications that were successfully discontinued at the pharmacy after being discontinued in the clinic. This change was sustained in the year following CancelRx (slope = 0.03 pp, 95% CI − 0.050 to 0.110) and did not revert to pre-CancelRx levels. The health IT functionality was able to effectively complete discontinuation tasks and potentially reduce workload for clinic staff.

**Conclusions:**

Overall, this study demonstrates the role that technology can play in promoting communication between clinics and pharmacies, especially when medications such as controlled substances are discontinued.

**Supplementary Information:**

The online version contains supplementary material available at 10.1186/s12911-022-01779-9.

## Background

In March 2018, the National Institute on Drug Abuse reported that more than 120 people die in the United States every day due to opioid overdoses [1]. The Centers for Disease Control and Prevention (CDC) cites this as a “serious national crisis” and estimates that the total economic burden of prescription opioid misuse amounts to $78.5 billion in the United States each year (including the cost of healthcare, lost productivity, addiction treatment, and criminal justice system involvement) [2]. More recent data from the CDC’s National Vital Statistics Report in December 2018 stated that the number of annual drug overdose deaths has increased 54% between 2011 and 2016 (increasing from 41,340 to 63,632 deaths per year) [3]. The CDC reported that the top 10 drugs involved in these overdose deaths belonged to three drug classes:

- Opioids: fentanyl, heroin, hydrocodone, methadone, morphine, and oxycodone
- Benzodiazepines: alprazolam and diazepam
- Stimulants: cocaine and methamphetamine.

In light of this crisis, agencies including state governments are promoting and requiring the use of electronic prescribing (e-prescribing) of all controlled prescriptions to reduce dispensing errors as well as drug diversion and misuse [4, 5]. When a medication is prescribed for a patient, a hand-written prescription creates opportunities for errors, such as incorrect transcription from the clinic electronic health record (EHR) to the prescription or incorrect translation from the prescription to the pharmacy system or vial label [4]. Additionally, a hand-written controlled substance prescription provides opportunities for individuals to tamper with or alter the contents from the prescriber’s original intent. Such alterations can include changing the strength, dose, quantity, or directions for use [6]. Electronic prescribing of controlled substances streamlines and controls all aspects of the drug dispensing process—sending the prescription directly from the prescriber’s EHR to the pharmacy dispensing software and minimizing the opportunities for unintentional error or intentional abuse.

However, e-prescribing also creates vulnerabilities in the dispensing of controlled substances. For example, federal law prohibits the inclusion of refills on prescriptions for Schedule II controlled substances (those deemed as having a currently accepted medical use but a high potential for abuse) [7]. The need for new prescriptions for these continuous orders may yield the same medication/strength/dose being listed several times on a patient’s medication list (both in the clinic EHR and pharmacy dispensing software). These duplicate orders may be confusing to decipher or add unnecessary noise throughout the prescribing and dispensing process. These vulnerabilities are further exacerbated when the dose of the medication is changed, resulting in confusion about which order is the most recent among a long list of the same product. These changes, as well as a general lack of integration, often lead to medication list discrepancies between clinic EHR and pharmacy dispensing platforms [8–10].

In addition to vulnerabilities in the ordering and dispensing processes, e-prescribing of controlled substances (EPCS) also presents unanticipated opportunities for drug diversion, misuse, and potential abuse. A documented anecdote describes a scenario in which a patient is seen at the clinic and is prescribed a controlled substance [11–13]. Knowing that the prescriber will transmit a prescription electronically to their desired pharmacy, the patient has a colleague stationed at the pharmacy to retrieve the medication as soon as it is filled. However, after the prescription is sent to the first pharmacy, the patient exclaims that they have changed their mind and requests that the order, instead be sent to another pharmacy (often a different chain or network than the first). After the second prescription is sent, they either have another colleague waiting there, or go to retrieve the medication themselves. Patients may avoid the Prescription Drug Monitoring Program (PDMP) flags or warnings at the pharmacy by filling these duplicate prescriptions the same day (pharmacies report to the PDMP every 24 h) [14, 15]. Similarly, patients may and avoid insurance billing errors or flags by paying cash or utilizing coupons. In these scenarios, even if the prescriber delegates a clinic staff member to contact the first pharmacy to discontinue the original prescription, it may be too late when the patient’s representative is already there to retrieve the medication as soon as it is filled.

These vulnerabilities emphasize the need for rapid and accurate communication of medication discontinuations between prescriber clinics and community pharmacies. There are various ways in which these medication discontinuations can be communicated, including notes attached to e-prescriptions, clinic staff telephone calls, faxed messages, or novel health information technology (health IT) such as CancelRx. CancelRx is a health IT functionality that utilizes the same electronic pathway as e-prescriptions [16–23]. A third party vendor (SureScripts) sends a communication directly from the prescriber’s EHR to the pharmacy dispensing software, but instead of authorizing the fill of a medication, it details its discontinuation. Depending on the community pharmacy integration, a CancelRx message can even identify and halt the processing of a previously e-prescribed prescription, working behind the scenes to ensure a prescription is effectively discontinued. In a pilot study, Pitts et al. demonstrated that CancelRx functionality successfully discontinued medications in the pharmacy’s dispensing software that had been discontinued in the prescriber’s EHR 92.4% of the time [23]. Similarly, a study by Chui et al, showcased not only a 93% success rate for CancelRx communication medication discontinuations, but also a drastic reduction in the amount of time required for a medication to be discontinued in the pharmacy software after it was discontinued at the clinic [24].

CancelRx has an added benefit, not just for patient safety and minimizing medication list discrepancies but thwarting the potential dispensing of prescription medications used for non-medical purposes. This presents an opportunity to assess the impact of CancelRx on the discontinuation and dispensing of prescription drugs that may be contributing to the opioid crisis. By electronically communicating medications that are discontinued in the physician’s office, this health IT functionality can potentially minimize the number of extraneous or unnecessary controlled medications dispensed and available to patients as well as reduce potential for diversion. This analysis is timely and relevant given CancelRx’s inclusion in CMS Meaningful Use Criteria (implemented in 2021) as well as ongoing need to address the serious opioid crisis [16, 18].

### Objectives

The main objective of the study was to measure the impact of CancelRx on reducing controlled substance medication list discrepancies between the clinic EHR and the pharmacy management software, specifically opioids, benzodiazepines, and stimulants (hereby referred to “controlled substances”). This objective was assessed by evaluating the percentage of controlled substance prescriptions successfully discontinued in the EHR and pharmacy management software over time. A secondary study objective was to compare the impact of CancelRx on reducing medication list discrepancies for controlled substances and non-controlled substances. This objective evaluated the percentages of controlled substance and non-controlled substance prescriptions successfully discontinued in the EHR and pharmacy management software over time. Finally, a third objective was to assess the impact of CancelRx on the length of time (in days) between controlled substance medication discontinuation in the clinic EHR and discontinuation in the pharmacy dispensing software.

## Methods

### Context

This study capitalized on the opportunity for a natural experiment, when an academic health system, UW Health in Wisconsin implemented CancelRx in October of 2017. Prior to CancelRx, when a prescriber decided to discontinue a medication, they would document the change in the clinic EHR. The prescriber could potentially send a message to the pharmacy documenting the medication change (call, fax, or e-prescription note), or delegate the task to an appropriate member of the clinic staff (often through EHR inbox messaging), but it was not required. The clinic staff could then follow through with communicating the discontinuation message to the pharmacy. Once a discontinuation message was received at the pharmacy, the pharmacy staff was responsible for identifying, discontinuing, and documenting the appropriate prescription. This process provided numerous opportunities for human or technology vulnerabilities that yielded incomplete discontinuation messages.

With CancelRx, when a prescriber decides to discontinue a medication, they still document the change in the EHR. However, for medications e-prescribed in the EHR, during the process of documentation the prescriber can relay the discontinuation information directly to the pharmacy via a CancelRx. Within UW Health, a prescriber can still opt out of sending a discontinuation message to the pharmacy, but the default/nonresponse setting is that a CancelRx is sent. A third-party vendor, SureScripts, then attempts to send the discontinuation message from the prescriber’s EHR to the community pharmacy dispensing system. SureScripts follows the same electronic signature used at the time of original prescribing to automatically identify the prescription in the pharmacy’s dispensing software and so that the system can halt it from being further dispensed or processed. Additionally, if a CancelRx message cannot be matched automatically within the pharmacy’s dispensing system to the appropriate prescription, a member of the pharmacy staff can manually match the discontinuation message to the appropriate prescription to ensure that it is discontinued within the system. At UW Health, if SureScripts is unable to send the discontinuation message electronically, it routes a message back to the appropriate clinic staff to follow-up with the necessary pharmacy manually. This near-instantaneous process allows for clinic staff to be informed of issues throughout the discontinuation process. This type of notification also alerts the pharmacy staff to changes in a patient’s medication list in addition to the immediate behind-the-scenes removal of the prescription.

### Data collection

UW Health underwent a phased approach of e-prescribing Schedule II medications that was completed prior to CancelRx implementation. Data extracted from the EHR included information regarding the patient (only those 18 +), clinic encounter, discontinued drug, and dates of discontinuation. Specific drug information captured from the EHR included the medication name, strength, and dosage form (e.g., Adderall XR 10 mg Capsule), pharmaceutical class (e.g., Stimulants), pharmaceutical subclass (e.g., Amphetamines), therapeutic class (e.g., Central Nervous System Drugs), and schedule (e.g., 2). Controlled substances included in the study were those identified drugs with a schedule 2, 3, 4, or 5 as defined by the United States Drug Enforcement Administration (DEA) and one of the following classifications:

- Pharmaceutical class: stimulant
- Pharmaceutical subclass: benzodiazepine, opioid agonist, opioid combination, or opioid partial agonist [25].

Because the health system in the study implemented mandatory e-prescribing of controlled substances, the research team was able to capitalize on the SureScripts e-prescription electronic identification to match medications that were discontinued in the clinic and supposed to be discontinued in the pharmacy both before and after CancelRx implementation. In addition to the use of the e-prescription signature, orders were also matched on patient gender, drug description (name, strength), ordering department, and a drug discontinuation time at the pharmacy within 72-h of when it was discontinued at the clinic.

The research team extracted data from the health system EHR regarding controlled substance medications discontinued in the outpatient clinics for 12-months prior to CancelRx implementation and for 12-months post implementation (allowing for a 4-week a priori burn-in period).

### Data analysis

An interrupted time series analysis (ITSA) was used to determine the impact of CancelRx on controlled substance medication list discrepancies over time. Specifically, the ITSA allowed the research team to assess the percentage of controlled substances that were discontinued in the clinic EHR and successfully discontinued in the pharmacy dispensing software within 72-h.

#### Single interrupted time series analysis (ITSA)

For the single ITSA, the study utilized Prais-Winsten estimation. The Prais–Winsten estimation is a procedure meant to take care of the serial correlation of type AR(1) in a linear model. The model may be written as:

$${Y}_{t}= {\beta }_{0}+ {\beta }_{1}{Time}_{t}+ {\beta }_{2}{X}_{t}+ {\beta }_{3}{X}_{t}{Time}_{t}+ {\beta }_{4}{discontinuations}_{t}+ {e}_{t}$$

Y_t_ is the aggregated percent of discontinuations for overall controlled substances measured at each equally spaced time point t (weekly), Time_t_ is the time since the start of the study, X_t_ is a dummy (indicator) variable representing the implementation of CancelRx (pre-intervention periods 0, otherwise 1), and X_t_Time_t_ is an interaction term. In the case of the single-group study, β0 represents the predicted intercept or starting level of the outcome variable. β1 is the slope or trajectory of the outcome variable until the introduction of the intervention. β2 represents the change in the level of the outcome that occurs in the period immediately following the introduction of the intervention (compared with the counterfactual). β3 represents the difference between pre-intervention and post-intervention slopes of the outcome. This study also incorporated adjusting covariates for the number of discontinuations per week (denominator) as β4. Thus, the study assessed significant p-values in β2 to indicate an immediate treatment effect, or in β3 to indicate a treatment effect over time. Test of autocorrelation using a procedure suggested by Cumby and Huizinga (1990, 1992) indicate than an autoregressive (AR1) was appropriate. Analysis was conducted in STATA [26–29]. A 1-week period constituted a measurement in the time series. 

#### Multiple interrupted time series analysis (ITSA)

In addition to the overall ITSA, the research team also compared the percentage of successful medication discontinuations for controlled substances and non-controlled substances.

#### Time-to-discontinuation

Furthermore, time-to-discontinuation event analyses were also conducted to compare the length of time between EHR and pharmacy system discontinuation before and after CancelRx implementation. The time to discontinuation was aggregated and averaged for each week in the study period and compared over time using regression with Newey-West standard errors [30, 31].

This study was approved by the University of Wisconsin-Madison Institutional Review Board.

## Results

**Fig. 1:**
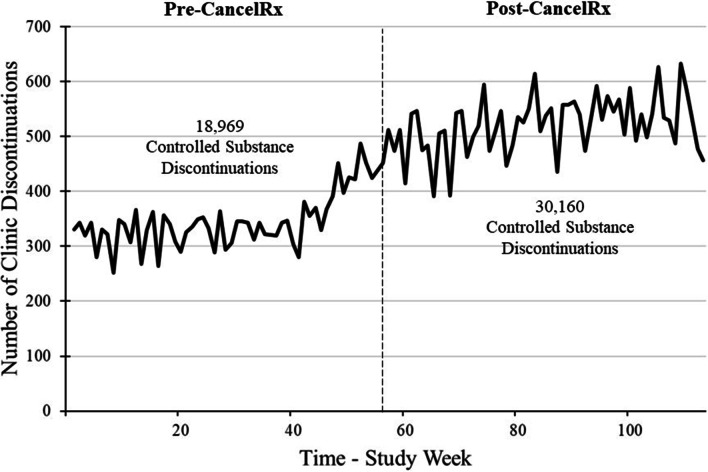
Controlled substance discontinuations over time. Prior to CancelRx implementation 18,969 controlled substances were discontinued at the clinic. After CancelRx implementation, 30,160 controlled substances were discontinued by providers and/or clinic staff

During the entire data collection period, 49,129 controlled substance medications were discontinued at the clinic, originating from UW Health providers and staff members. In the year prior to CancelRx implementation, 18,969 controlled substance medications were discontinued with over half of the medications classified as Schedule II drugs (14,627, 77%). After CancelRx implementation, 30,160 controlled substance medications were discontinued and 80% were Schedule II drugs (24,323). In comparison, 354,690 non-controlled medications were discontinued by the clinics during the same study period. Figure 1 details the controlled substance medication discontinuations per week.

### Outcome 1: percentage of successful controlled substance discontinuations over time

**Fig. 2:**
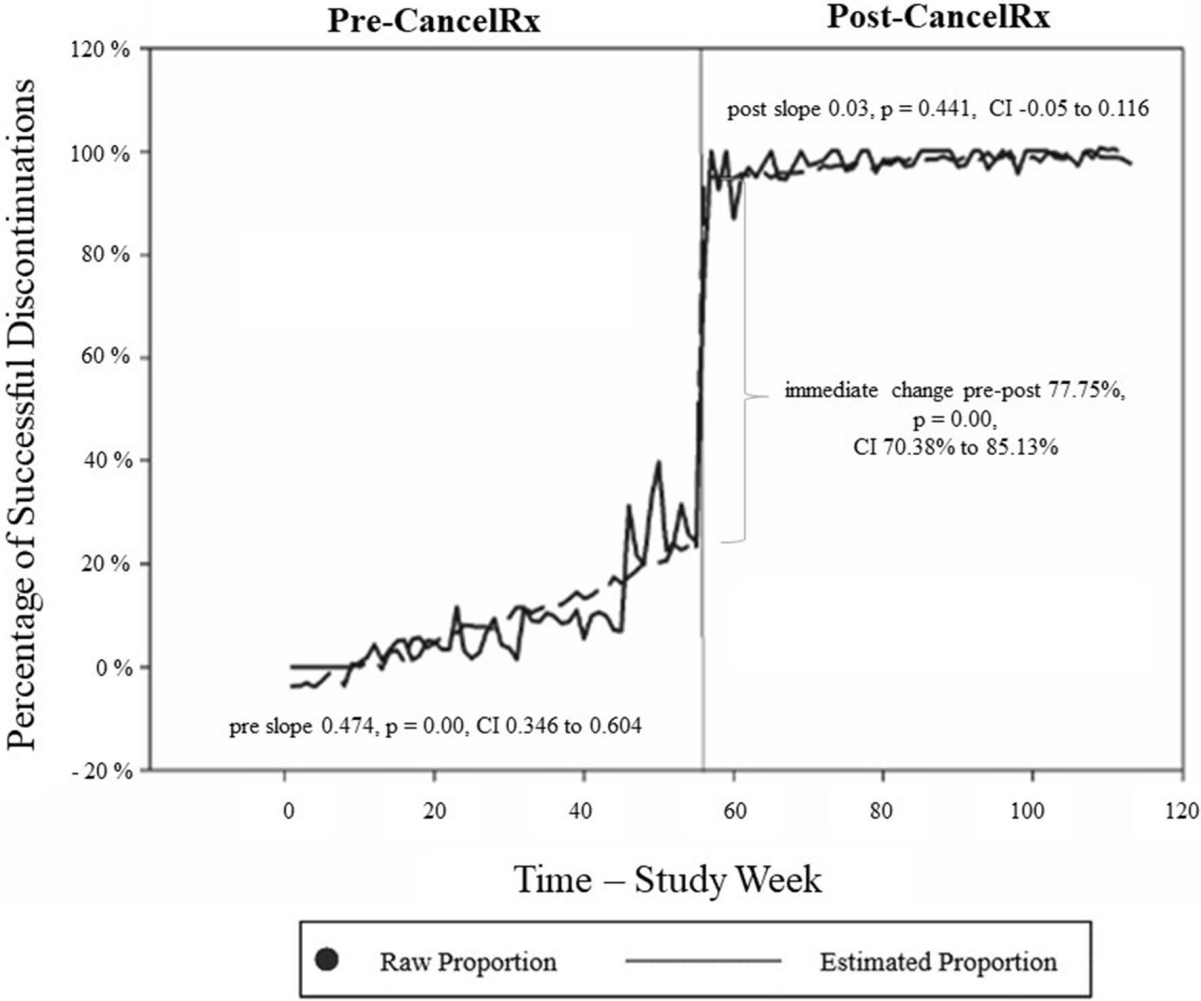
Successful controlled substance medication discontinuations over time. Immediately following CancelRx implementation, there was a 77.75 percentage point increase in the percentage of medications successfully discontinued in both the clinic EHR and pharmacy dispensing systems

Figure 2 illustrates the ITSA for all controlled substances, where the x-axis depicts time (in weeks) with a vertical line at Week 56 indicating CancelRx implementation. The y-axis depicts the percentage of medications that were discontinued successfully, meaning those medications discontinued in both the clinic and pharmacy systems (out of all medications discontinued by the clinic). The pre-intervention period showed a moderate trend (i.e., slope) increase prior to implementation of CancelRx (0.474 percentage point [pp] increase per week, 95% CI 0.346 to 0.603). After CancelRx implementation, there was an immediate and significant increase in the percentage of successful medication discontinuations, an increase of 77.7 pp (adjusted for discontinuations per week, p < 0.001). The post-implementation slope remained stable at 0.03 pp (95% CI − 0.050 to 0.110) and did not decline to pre-CancelRx levels. Prais-Winsten estimation and equation terms are presented in Table 1.

**Table 1:** Overall interrupted time series analysis

	Coef	Std. Err	t	P >|t|	95% CI	
Pre-intervention trend (β1)	0.474	0.065	7.29	< 0.001	0.34559	0.6035
Immediate effect (β2)	77.754	3.7191	20.91	< 0.001	70.382	85.1262
Difference in pre and post trends (β1 + β3)	− 0.4424	0.07246	− 6.11	< 0.001	− 0.586	− 0.298
Post-intervention trend (β3)	0.03	0.04	0.77	0.441	− 0.05	0.11
Adjusting covariate (β4) number of weekly discontinuations	0.0608	0.0278	2.18	0.031	0.00562	0.116
Predicted initial level (Intercept β0)	− 10.667	3.062	− 3.48	0.001	− 16.7364	− 4.597

### Outcome 2: percentage of successful discontinuations over time for controlled substances and non-controlled substances

**Fig. 3:**
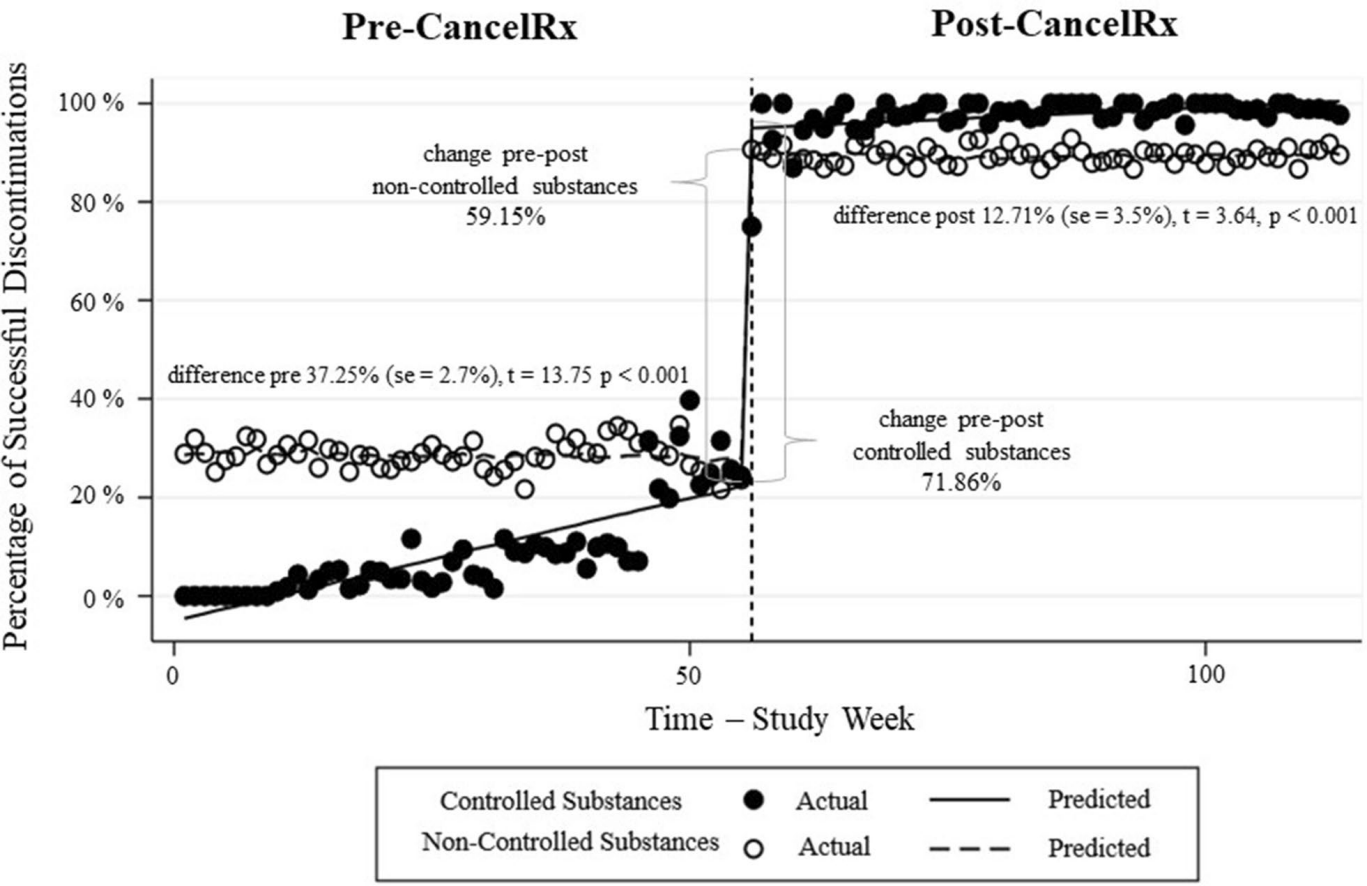
Successful controlled substance and non-controlled substance medication discontinuations over time. Prior to CancelRx, non-controlled substances had a higher percentage of successful discontinuations than controlled substances. After CancelRx, controlled substances demonstrated a slightly higher percentage

Figure 3 illustrates the ITSA comparing the percentage of successful medication discontinuations for controlled substances compared to non-controlled substances. The ITSA illustrated that, while the trend for non-controlled substances remained fairly stable in the year prior to CancelRx implementation (pre-implementation slope − 0.02 pp, 95% CI − 0.082 to 0.046), the percentage of successful controlled substance discontinuations gradually increased until almost converging at approximately 30% success.

Immediately following CancelRx implementation, the percentage of successfully discontinued medications significantly increased for both controlled and non-controlled substances (71.86 pp and 59.15 pp respectively). However, the increase was greater for controlled substances than non-controlled substances, and this difference persisted throughout the year following CancelRx implementation. The trends for both controlled substances and non-controlled substances were stable in the post-CancelRx implementation period. The multiple ITSA estimation parameters can be found in Additional file 1 (Table S1 and Table S2).

### Outcome 3: time to discontinuation of controlled substances between clinic and pharmacy over time

The third outcome of the study compared the average amount of time between when a controlled medication was discontinued in the clinic EHR and when it was discontinued in the pharmacy dispensing software.

**Fig. 4:**
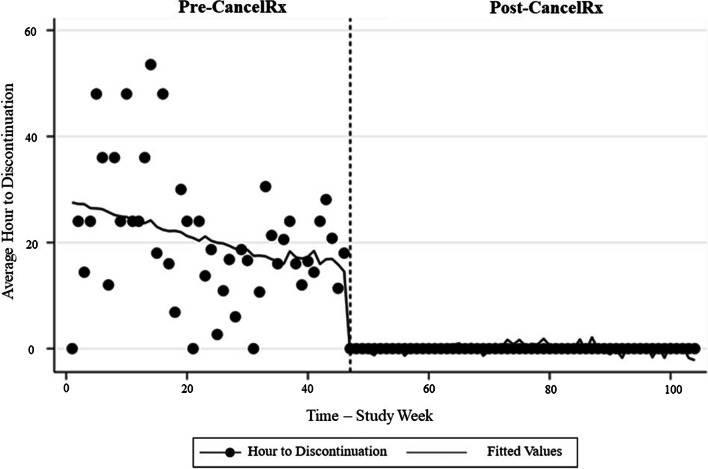
Average time to controlled substance discontinuation over time. The average time (in hours) between when a medication was discontinued at the clinic to when it was discontinued at the pharmacy decreased after CancelRx implementation

Given that the clinic EHR data contained both date and time stamps for when a medication was discontinued, whereas the pharmacy data contained only the date, it was necessary to address this inconsistency. As such, prescriptions that were discontinued on the same day (in both the clinic and pharmacy) were coded as “0 days” (indicating 0 days difference between the two datasets). Similarly, discontinuations that occurred in the pharmacy the following day were coded as 1 day. When aggregated to yield the average weekly results, the data often yielded fractions of days. To simplify the results, Fig. 4 is presented as hours (i.e., 0.5 days is presented as 12 h), even though data was not collected at the level of the hour. The time required for a medication to be discontinued in the pharmacy after it had been discontinued at the clinic varied during the year prior to CancelRx implementation. Using regression with Newey-West standard errors, the time to discontinuation was 27.43 h (se = 4.48, t = 6.12, p < 0.001, CI 18.53—36.31). Prior to CancelRx implementation, there were several weeks in which all orders were discontinued the same day (represented with y-values = 0) or weeks in which the average time was over 2 days (y-values > 48). The predicted trend-line gradually decreased in the weeks prior to CancelRx implementation.

After CancelRx implementation, medication discontinuations were all completed on the same day (all values were = 0) with a stable trend and almost no variation.

## Discussion

Overall, implementation of CancelRx showed a marked improvement in the percentage of medications that were successfully discontinued in the pharmacy after being discontinued at the clinic. The addition of this novel technology effectively minimized the incidence of medication list discrepancies at the pharmacy. However, the study results must be situated in the environmental context of October 2017 when CancelRx was implemented.

### Pre-CancelRx

The pre-intervention period demonstrated a gradual increase in the number of medications that were successfully discontinued in both the clinics and pharmacies leading up to CancelRx implementation. There were several organization-wide factors that may have contributed to this upward trend. First, in the year prior to CancelRx, UW Health began incrementally implementing the electronic transmission of controlled substance prescriptions to pharmacies (specifically EPCS). Although prescriptions had long before been “electronically” documented in the clinic’s EHR, prior to EPCS prescribers would print-out and hand the patients a paper copy of their prescription or mail prescriptions to the pharmacy to be dispensed. Prior to EPCS, clinic staff may have faced difficulty tracking exactly where a patient filled their prescription. This could lead to confusion regarding who clinic staff should notify when medications were stopped or discontinued. Even if clinic staff members attempted to contact the patient’s preferred pharmacy (as documented in the EHR), the patient could have filled the prescription elsewhere and the pharmacy staff would have no record of the medication being discontinued.

As the health system implemented EPCS in the year prior to CancelRx, clinic staff could ensure where prescriptions were being filled (based on where they were electronically sent) and, therefore, contact the pharmacies with the medication on record. Additionally, the EPCS implementation may have facilitated a culture shift and discussion surrounding Schedule II controlled substances that contributed to increased staff awareness and ensuring the need to communicate when medications were stopped or discontinued. For example, when prescriptions can be electronically transmitted to the pharmacy via the patient’s EHR, there is potentially more importance on having a concise, clean, and up-to-date medication list without duplicate or outdated prescriptions. This “clean” list would ensure that the most recent and correct prescriptions are renewed. An organizational culture emphasizing the importance of up-to-date medication lists may lead clinic staff to increasingly contact the pharmacy regarding outdated or previously discontinued prescriptions.

Beyond the scope of the singular UW Health organization, Wisconsin enacted legislation that required prescribers and clinic staff to check the Prescription Drug Monitoring Program (PDMP) prior to writing a prescription for a controlled substance [32]. Occurring in April 2017, this statute emphasized the use of the statewide registry that documents the patient, drug name, drug strength, quantity and pharmacy information, as well as third-party payment methods used when opioids, benzodiazepines, and stimulants are filled. Mandated use of this registry may have heightened awareness surrounding the prescribing and discontinuation of controlled substances and led to the increasing trend in the months preceding CancelRx implementation.

**Fig. 5:**
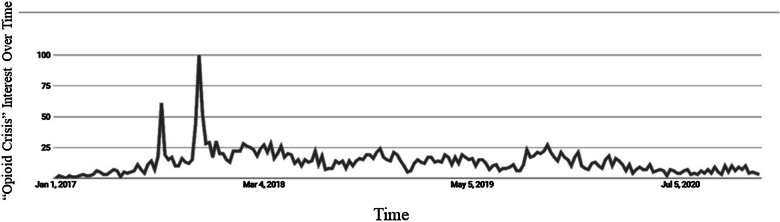
“Opioid epidemic” interest over time. Data Source: Google Trends. Within the figure, numbers represent search interest relative to the highest point on the chart for the given region and time. A value of 100 is the peak popularity for the term, approximately October 2017, which coincides with CancelRx implementation

The timeline leading up to CancelRx implementation at UW Health in October 2017 also coincides with events on a national scale. On October 26, 2017, the President of the United States declared the opioid crisis a public health emergency. This public declaration led to an increase in public awareness surrounding opioid and controlled substance prescribing, as evident by the Google Trend line shown in the Fig. 5 (indicating the number of times the term “opioid crisis” was searched over time). Once again, this announcement likely increased general awareness of controlled substance prescriptions, and motivated clinics, prescribers, and pharmacies alike to have accurate and up-to-date medication lists for patients using these medications.

### Post-CancelRx

After CancelRx implementation, there was a significant increase in the communication of medication cancellation messages. Although the influence of local, state, and national environment surrounding controlled substances is still important to consider, the drastic and sustained success also seems attributable to the CancelRx functionality itself. CancelRx was designed to work “behind the scenes” and automate the previously manual process, eliminating the need for clinic staff to intervene, except in cases in which the prescription was not electronically transmitted to the pharmacy (e.g., prescriptions written prior to EPCS), the CancelRx message could not be sent (e.g., the pharmacy had not turned on the functionality) or the pharmacy was unable to find a match for the discontinued prescription (e.g., the prescription had been transferred out to another pharmacy). This demonstrated that not only was the health IT functionality able to take over tasks and reduce workload for clinic staff, but that they were able to perform the task more rapidly.

CancelRx impacted the way that clinics and pharmacies communicated regarding the discontinuation of controlled substance medications. CancelRx may have also improved coordination and communication amongst prescribers and within the clinics themselves. Implementation of the CancelRx functionality may have increased prescriber and clinic awareness and motivation to address communication of controlled substance discontinuations. The ability to discontinue medications quickly and reliably from a patient’s medication list in the EHR may have prompted prescribers and clinic staff to “clean up” patient’s profiles, ensuring that only the most recent and up-to-date medications were listed on the patient’s records. CancelRx functionality and implementation possibly helped to facilitate a common organizational mental model around the way that controlled substance medications should be documented, especially when documenting medication discontinuations [33, 34].

Another way in which CancelRx may have improved communication and reduced barriers within the clinic is when prescribers are requested to “cover” for their colleagues by prescribing continuing doses of controlled substances when the original prescriber is unavailable. If a patient or patient’s pharmacy requests a refill requested of a controlled substance, UW Health policy indicated that the clinic staff should first verify that the patient is eligible for a refill by checking the PDMP and noting the last fill date of the prescription. If the patient is due for a refill, the clinic staff sends the request off to the patient’s provider or original prescriber. If the provider is not in the clinic, the request may be forwarded to another prescriber in the clinic who is “covering” for the intended recipient. This process exposes the patient to potential vulnerabilities. The first potential vulnerability occurs when the patient’s medication list in the EHR still has a medication on the patient’s profile that was actually discontinued. The clinic staff and prescriber may look at the patient’s chart and not know or see that the original prescriber actually wanted the patient to stop taking the medication. Unknowingly, the covering prescriber could continue the medication therapy by authorizing refills. In this case, CancelRx and the awareness and ease of ensuring an up-to-date and accurate medication lists, likely reduces the unintended supply of controlled substances reaching patients. A second potential vulnerability occurs when the patient’s care plan includes tapering the doses down of controlled substance medications. The clinic’s covering provider may not know or be able to find documentation of the plan in the patient’s EHR to slowly reduce the patient’s dose or directions and simply continue the patient’s previous therapy. CancelRx, and the clinic's organizational mental model surrounding discontinued medications facilitates safe patient handoffs between providers.

### Impact of CancelRx on controlled substances and non-controlled substances

In comparing the results of the controlled substances and non-controlled substances post-CancelRx the overall levels of successful discontinuations for the controlled substances were higher than that of the non-controlled substances. This may be in part to the organizational, state, and national emphasis placed on controlled substance prescribing (and discontinuation). This perhaps confirms the notions of a shared mental model and organizational and cultural awareness surrounding this issue and the desire to minimize unintentional supply of controlled substances reaching patients. To put it another way, prescribers and clinic staff may have been motivated to ensure up-to-date medication lists in the post-CancelRx period not only because of the priority and attention given to the topic but also because the functionality reduced barriers to communication.

### Limitations

A study must be considered in light of its limitations. This study has limited generalizability because it occurred within a single health system. Additionally, this health system included outpatient clinics and affiliated community pharmacies, which had access to a patient’s EHR to further investigate questions regarding medication discontinuations or patient’s care plans. Future studies should assess the impact of CancelRx for patients who fill prescriptions at community pharmacies outside of a singular health system to determine the impact on medication list discrepancies and communication between prescribers and pharmacists.

A second limitation is the confounding effects of the environment in which CancelRx implementation took place. Situated amongst ECPS roll outs, PDMP statues, and attention at the national level, the effects of CancelRx may be inflated. However, the results are still convincing given the drastic and sustained increase in medication discontinuations communicated after CancelRx implementation.

## Conclusions

This study demonstrated the impact of a novel technology, CancelRx, on communicating medication discontinuations between clinics and pharmacies. Namely, this study assessed the changes in medication list discrepancies when the prescriptions being discontinued were controlled substances. Considering the opioid epidemic, it is crucial for both clinics and pharmacies to have up-to-date medication lists, not only for the safety of patients but also to prevent abuse and misuse within their communities. This study demonstrates the role that technology can play in promoting controlled substance medication safety.

## Supplementary Information

**Additional file 1.** Multiple ITSA Parameters. Multiple Interrupted Time Series Analysis (ITSA) parameters for successful controlled substance and non-controlled substance medication discontinuations over time. Additional File 1 contains two tables: **Table S1**. Multiple ITSA—Configuration 1 and **Table S2**. Multiple ITSA—Configuration 2 (flipped).

## Data Availability

The data underlying this article cannot be shared publicly for the privacy of individuals that participated in the study; the dataset contains protected health information. The data underlying this article were provided by UW Health by permission. Data will be shared on reasonable request to the corresponding author with permission of UW Health.
