# Supplementary material for: Impact of CancelRx on discontinuation of controlled substance prescriptions: an interrupted time series analysis
Source: BMC Med Inform Decis Mak. 2022 Feb 25;22:50. doi: 10.1186/s12911-022-01779-9 (PMC8876377; doi:10.1186/s12911-022-01779-9)
Supplement: Supplementary file 1 — Additional file 1. Multiple ITSA Parameters. Multiple Interrupted Time Series Analysis (ITSA) parameters for successful controlled substance and non-controlled substance medication discontinuations over time. Additional File 1 contains two tables: Table S1. Multiple ITSA—Configuration 1 and Table S2. Multiple ITSA—Configuration 2 (flipped). [file 12911_2022_1779_MOESM1_ESM.docx]

**Additional File 1 – Multiple ITSA Parameters**

The following two tables and figure detail the Multiple Interrupted Time Series Analysis (ITSA) parameters for successful controlled substance and non-controlled substance medication discontinuations over time.

***Supplementary Table 1.*** ***Multiple ITSA – Configuration 1***

|  | Coef. | Std. Err. | t | P > \|t\| | 95% CI |
| --- | --- | --- | --- | --- | --- |

| Adjusting covariate | -0.00337 | 0.0019 | -1.71 | 0.089 | -0.00725 | 0.000514 |
| --- | --- | --- | --- | --- | --- | --- |
| Pre-intervention trend for non-controlled substances | -0.01815 | 0.0326 | -0.56 | 0.579 | -0.08245 | 0.04615 |
| Pre-intervention difference in intercepts | -37.25341 | 2.7084 | -13.75 | <0.001 | -42.59163 | -31.9152 |
| Pre-intervention difference in slopes | 0.51711 | 0.0758 | 6.81 | <0.001 | 0.36753 | 0.66669 |
| Change in level for non-controlled substance | 59.15015 | 1.7541 | 33.72 | <0.001 | 55.6928 | 62.6075 |
| Pre-intervention trend for controlled substances | 0.49896 | 0.07617 | 7.29 | <0.001 | 0.364 | 0.6339 |
| Post-intervention immediate difference between controlled substance and non-controlled substances | 12.70645 | 3.4947 | 3.64 | <0.001 | 5.818 | 19.5945 |
| Post-intervention trend for controlled substance | 0.1 | 0.04 | 2.68 | 0.007 | 0.03 | 0.17 |
| Post-intervention trend for non-controlled substance | 0.02 | 0.01 | 1.135 | 0.257 | -0.01 | 0.05 |
| Post-intervention trend difference | 0.08 | 0.04 | 2.07 | 0.04 | 0.0038 | 0.159 |
| Difference pre-intervention versus post-intervention non-controlled substance trend | 0.0351 | 0.0368 | 0.95 | 0.342 | -0.037 | 0.107 |
| Difference pre-intervention versus post-intervention controlled substance trend | -0.40041 | 0.08266 | -5.22 | <0.001 | -0.551 | -0.249 |
| Initial level (intercept) for Non-controlled substance | 33.04919 | 2.56687 | 12.88 | <0.001 | 27.99 | 38.1 |

***Supplementary Table 2.*** ***Multiple ITSA – Configuration 2 (flipped)***

|  | Coef. | Std. Err. | t | P > \|t\| | 95% CI | |
| --- | --- | --- | --- | --- | --- | --- |
| Adjusting covariate | -0.00337 | 0.001 | -1.71 | 0.089 | -0.00725 | 0.000514 |
| Pre-intervention trend for controlled substances | 0.49896 | 0.068 | 7.29 | <0.001 | 0.364 | 0.63392 |
| Pre-intervention difference in intercepts | 37.25341 | 2.708 | 13.75 | <0.001 | 31.9152 | 42.59163 |
| Pre-intervention difference in slopes | -0.51711 | 0.075 | -6.81 | <0.001 | -0.66669 | -0.36753 |
| Change in level for controlled substances | 71.8566 | 3.066 | 23.43 | <0.001 | 65.8129 | 77.90031 |
| Pre-intervention trend for controlled substances | -0.01815 | 0.032 | -0.56 | 0.57 | -0.0824 | 0.04615 |
| Post-intervention immediate difference between controlled substance and non-controlled substances | -12.706 | 3.494 | -3.64 | <0.001 | -19.594 | -5.8183 |
| Post-intervention trend for non-controlled substances | 0.02 | 0.01 | 1.135 | 0.25 | -0.01 | 0.05 |
| Post-intervention trend for controlled substance | 0.1 | 0.04 | 2.689 | 0.007 | 0.03 | 0.17 |
| Post-intervention trend difference | -0.0816 | 0.039 | -2.07 | 0.04 | -0.159 | -0.003 |
| Difference pre-intervention versus post-intervention controlled substance trend | -0.40041 | 0.076 | -5.22 | <0.001 | -0.551 | -0.249 |
| Difference pre-intervention versus post-intervention non-controlled substance trend | 0.0351 | 0.036 | 0.95 | 0.34 | -0.037 | 0.107 |
| Initial level (intercept) for controlled substances | -4.20423 | 1.3443 | -3.13 | 0.002 | -6.853 | -1.5546 |
